# The long noncoding RNA ASNR regulates degradation of Bcl-2 mRNA through its interaction with AUF1

**DOI:** 10.1038/srep32189

**Published:** 2016-08-31

**Authors:** Jiahui Chen, Lihui Liu, Guifeng Wei, Wei Wu, Huaxia Luo, Jiao Yuan, Jianjun Luo, Runsheng Chen

**Affiliations:** 1 Key Laboratory of RNA Biology, Institute of Biophysics, Chinese Academy of Sciences, Beijing 100101, China; 2 Beijing Key Laboratory of Noncoding RNA, Institute of Biophysics, Chinese Academy of Sciences, Beijing 100101, China; 3 University of Chinese Academy of Sciences, Beijing 100049, China; 4 Research Network of Computational Biology, RNCB, Beijing, 100101, China

## Abstract

The identification and characterization of long non-coding RNAs (lncRNAs) in diverse biological processes has recently developed rapidly. The large amounts of non-coding RNAs scale consistent with developmental complexity in eukaryotes, indicating that most of these transcripts may have functions in the regulation of biological processes and disorder in the organisms. In particular, Understanding of the overall biological significance of lncRNAs in cancers still remains limited. Here, we found a nuclear-retained lncRNA, termed Lnc_ASNR (apoptosis suppressing-noncoding RNA), which serves as a repressor of apoptosis. Lnc_ASNR was discovered in a set of microarray data derived from four kinds of tumor and adjacent normal tissue samples, and displayed significant up-regulation in the tumor tissues. Using an RNA-pull down assay, we found that Lnc_ASNR interacted with the protein ARE/poly (U)-binding/degradation factor 1(AUF1), which is reported to promote rapid degradation of the Bcl-2 mRNA, an inhibitor of apoptosis. Lnc_ASNR binds to AUFI in nucleus, decreasing the cytoplasmic proportion of AUF1 which targets the B-cell lymphoma-2 (Bcl-2) mRNA. Taken together, the overall effect of Lnc_ASNR expression is thus a decrease in cell apoptosis indicating that Lnc_ASNR may play a vital role in tumorigenesis and carcinogenesis.

With the great advance in high resolution microarray and whole-genome sequencing technologies in recent years, we now have markedly widened our vision of the mammalian transcriptomes field^1^,^2^,^3^. Dedicated consortiums, such as the GENCODE project, have shown that the majority of genomic DNA is transcribed while less than 2% of the DNA is finally translated into proteins^4^. The complexity of biological processes among species correlates better with the amount of expressed non-coding RNAs, which constitute the majority of human genomic transcripts than with the number of coding genes that are translated into proteins^5^. This suggests that non-coding RNAs may play an increasingly crucial role in the evolution of developmental complexity in eukaryotes.

According to transcript size, non-coding RNAs are classified into small non-coding RNAs(<200nt) and long non-coding RNAs (lncRNAs:>200nt)^6^, the latter frequently being, polyadenylated and devoid of evident open reading frames (ORFs)^4^. Compared with coding genes, lncRNAs tend to be less conserved across species and show higher tissue specificity^4^,^7^. lncRNAs have now been found to participate in a number of biological processes such as pluripotency maintenance, cell differentiation and proliferation, apoptosis and genomic imprinting via variable mechanisms at multiple levels, including chromatin modification, transcriptional and post-transcriptional processing^2^,^8^,^9^,^10^,^11^,^12^. A great many studies have indicated that lncRNAs are related to a wide variety of diseases, such as cancers, cardiovascular diseases and neurodegeneration diseases^10^,^13^,^14^,^15^. HOTAIR, one of the most well characterized lncRNAs, recruits the Polycomb Repressive Complex 2 (PRC2) to repress the HoxD genes. The PRC2 subsequently induces heterochromatin formation at specific gene loci, leading to inactivation of the targeted genes^16^. Moreover, the increased expression of HOTAIR in primary breast tumors negatively affects the prognosis of the patients^9^. The lncRNA PVT1 plays a role in G1 arrest through epigenetically regulating the expression of p15 and p16 by binding to enhancer of zeste homolog 2 (EZH2), and its expression is markedly increased in gastric cancer tissues^17^. The lincRNA-p21 is regulated by p53, suppressing cell apoptosis. It recruits hnRNP-K to p53 target genes to achieve the p53 mediated regulation. LincRNA-p21 has lately also been reported to be important in colorectal carcinoma^18^. The LncRNADisease database, integrates more than 1000 lncRNA-disease entries and 475 lncRNA interaction entries, thereby connecting data on 321 lncRNAs and 221 diseases from about 500 publications^19^. The above studies show that research on lncRNAs may further the understanding of diseases and malignancies, thus facilitating future innovation and advances in disease diagnosis, treatment and prognosis.

Messenger RNA (mRNA) turnover is a major factor in gene expression^20^,^21^. AU-rich elements (AREs) are a varied family of 3′-untranslated sequences often containing repetitive AUUUA or similar motifs recognized by a diverse population of trans-acting protein named ARE-binding proteins (AUBPs)^22^. Among the AUBPs, the ARE/poly (U)-binding/degradation factor 1 (AUF1), is one of the best characterized^23^. AUF1 is expressed as four protein isoforms generated by alternative splicing of a single pre-mRNA. The AUF1 proteins are named according to their apparent molecular masses as p37AUF1, p40AUF1, p42AUF1, and p45AUF1^24^. AUF1 increase the mRNA degradation rates by assembling factors necessary for RNA decay including translation initiation factor eIF4G, chaperones hsp27, hsp70 and PABP, etc.^25^,^26^.

In mammals, AUF1 regulates the expression of many key players in cancer including proto-oncogenes, anti-oncogenes, pro-inflammatory cytokines and regulators of apoptosis and the cell cycle^27^. Among these, the anti-apoptotic Bcl-2 protein is overexpressed in a variety of cancers where it enhances cell survival and can obstruct the efficacy of radiological and chemotherapeutic treatments of the cancer. Cytoplasmic AUF1 binds to the 3′UTR of the Bcl-2 mRNA and accelerates mRNA degradation^28^,^29^. A number of potentially anti-apoptotic lncRNAs, such as PCGEM1 and PANDA^30^,^31^, as well as pro-apoptotic lncRNAs, like lincRNA-p21, GAS5, and MEG3 have been reported, but few of them regulate the Bcl-2 apoptotic pathway like lncRNA CDIR^18^,^32^,^33^,^34^. Consequently, we were interested in studying the role of lncRNAs in the regulation of the anti-apoptotic gene Bcl-2.

AUF1 is predominantly a nuclear protein, but all isoforms shuttle between the nucleus and cytoplasm^35^,^36^. AUF1-dependent control of mRNA decay is most consistently associated with cytoplasmic AUF1 proteins^27^,^28^. Besides, nucleocytoplasmic shuttling is a common property of many RNA-binding proteins^37^. The subcellular distribution of AUF1 is highly dependent on isoform and can be modulated by cellular signaling pathways and other extrinsic stimuli. Moreover, the subcellular localization of AUF1 proteins is dysregulated in diverse cancers^27^,^38^. Several reports have shown that lncRNA completely binds AUF1 to release the target mRNA binding sites^34^,^39^. Besides, AUF1 modulates NEAT1 levels and localization in order to control the nuclear export of a specific set of NEAT1-target mRNAs whose localization is regulated by NEAT1^40^. However, to our knowledge, there exist no reports of lncRNAs in the translocation of AUF1 in cancer cells. Consequently, the possibility of lncRNAs being involved in the regulation of AUF1 nucleus/cytoplasmic compartment shuttle thus deserves attention.

Here, we report a nuclear-retained lncRNA, termed Lnc_ASNR (apoptosis suppressing-noncoding RNA), which serves as a repressor of apoptosis. Lnc_ASNR displays obvious up-regulation in four tumor tissues compared to matched normal surrounding tissues. Moreover, Lnc_ASNR was shown to interact with AUF1 in the nucleus and increased the nuclear proportion of AUF1. Consequently, the decreased cytoplasmic AUF1 level depressed AUF1 mediated Bcl-2 mRNA degradation. This resulted in high expression of Bcl-2.

## Results

### Identification and characterization of Lnc_ASNR in cancer

**Figure 1:**
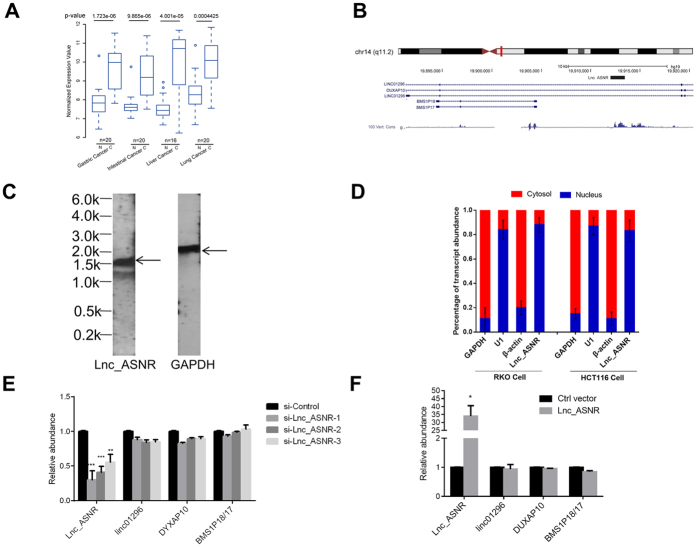
Identification and characterization of the long non-coding RNA Lnc_ASNR in cancer. (**A**) Expression of Lnc_ASNR in four types of paired tumor tissue samples by microarray. N represents normal tissue samples, and C represents cancer tissue samples. (**B**) Genomic location of Lnc_ASNR relative to upstream and downstream genes. (**C**) Northern blots of the Lnc_ASNR and GAPDH transcripts. (**D**) Quantification of Lnc_ASNR expression in fractionated RKO and HCT116 cell lysates by qRT-PCR. U1 RNA serves as a positive control for nuclear gene expression, and GAPDH and β-actin serve as controls for cytoplasmic gene expression. Error bars represent SEM, n = 3. (**E**) qRT-PCR of Lnc_ASNR and its host and neighboring genes in RKO cells after knockdown of Lnc_ASNR by three different siRNAs. (**F**) qRT-PCR of Lnc_ASNR and its host and neighboring genes in HCT116 cells after transfection with the Lnc_ASNR expression vector.

We recently carried out a transcriptome profiling analysis for the microarray data (NCBI GEO accession number GSE70880) of the four kinds of patient samples (gastric, colon, liver, and lung, paper accepted, DOI: 10.18632/oncotarget.6993). Among them, a novel lncRNA, termed as Lnc_ASNR, was identified as relatively high expression in all tumor samples (Fig. 1A). We validated the expression of the lncRNA in various cell lines, and the quantitative reverse transcription-polymerase chain reaction (qRT-PCR) results showing that the RKO and HCT116 cell lines displayed relatively high and low level, respectively, of Lnc_ASNR (Supplementary Fig. 1). Subsequently, we attempted to characterize the functions of Lnc_ASNR in RKO and HCT116 cell lines.

Lnc_ASNR is located in the chr14-q22.2 region. It has been annotated as ENST00000548057 in GENCODE Version 17 (Fig. 1B). After performing 5′ and 3′ RACE (rapid amplification of cDNA ends) assays in RKO cells, we found that the transcript is polyadenylated and has one exon with 1428nt mapping to position chr14: 19913257-19912166 (Supplementary Fig. 2). Northern blot analysis confirmed the transcript size of approximately 1.4 kb (Fig. 1C). We further assessed the coding potential of the transcript with PhyloCSF algorithm and obtained a coding probability of 13.3515 which suggests the transcript to be non-coding. Isolation of nuclear and cytoplasmic fractions in RKO cells and HCT116 cells indicated that the transcript was mainly located in the nucleus (similar to that of U1, Fig. 1D). As lncRNAs are frequently reported to act on its host gene and neighboring genes^41^,^42^, we test the relationship between Lnc_ASNR, its host gene lincRNA 01296 and its neighboring genes DYXAP10 and BMS1P18/17 to assess whether Lnc_ASNR possibly exerts its function *in cis*. We consequently knocked down the expression of Lnc_ASNR using three different siRNAs, and examined the expression level of lincRNA 01296 and mRNA levels of DYXAP10 and BMS1P18/17 by qRT-PCR. None of the genes show any significant difference in expression after knockdown of Lnc_ASNR (Fig. 1E). Neither did overexpressing Lnc_ASNR influence the expression of these genes (Fig. 1F). Thus, if functional, Lnc_ASNR does not appear to act *in cis* to regulate the expression of its host gene or its neighboring genes.

### Lnc_ASNR promotes cell proliferation and suppresses cell apoptosis

Since Lnc_ASNR exhibited higher expression levels in tumor samples than adjacent normal tissues, we determined to investigate the physiological role of Lnc_ASNR in tumorigenesis related processes.

**Figure 2:**
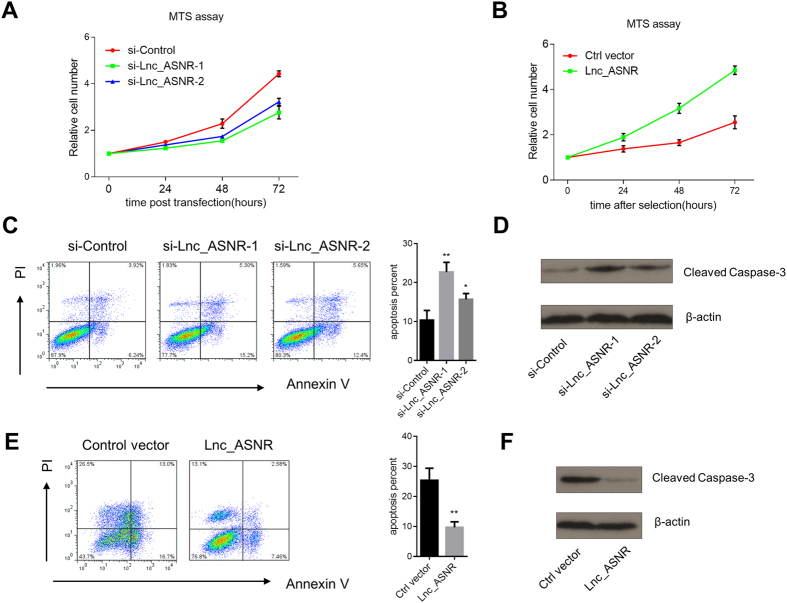
Lnc_ASNR promotes cell proliferation and suppresses cell apoptosis. (**A**) Absorbance Optical density (OD) at 490 nm in the MTS assay measured every 24 h after transfection with anti-Lnc_ASNR siRNAs in RKO cells. (**B**) Absorbance Optical density (OD) at 490 nm in the MTS assay every 24 h after transfection with pcDNA3.1-Lnc_ASNR in HCT116 cells (**C**) Cell apoptosis ratio after Lnc_ASNR knockdown measured by flow cytometry in RKO cells. The X-axis represents Annexin-V and the Y-axis PI staining. The bar plot on the right gives the results from quantification (% apoptotic cells) average of three independent replicate experiments. Bars indicate SEM. *P < 0.05. **P < 0.01. (**D**) Increase in Caspase 3 cleavage 48 h after Lnc_ASNR knockdown by siRNA transfection in RKO cells. (**E**) Cell apoptosis after Lnc_ASNR overexpression measured by flow cytometry in HCT116 cells. For further details, see 2C (above) (**F**) Increase in Caspase 3 cleavage 24 h after transfection of HCT116 cells with the Lnc_ASNR expression vector.

First, we performed the MTS assay to monitor the effect of Lnc_ASNR on cell proliferation. We observed a significant decrease in proliferation of cells treated with siRNAs targeting Lnc_ASNR compared with negative control in RKO cells (Fig. 2A). Furthermore, overexpression of Lnc_ASNR in HCT116 cells resulted in a significant increase in cell proliferation (Fig. 2B). These results suggest that Lnc_ASNR plays a role in regulating cell proliferation. However, the detailed mechanisms by which the lncRNA functions warrants further investigations.

We then tested the effect of Lnc_ASNR on the cell cycle arrest by fluorescence-activated cell sorting (FACS) analysis. Knockdown and overexpression of Lnc_ASNR both showed no significant changes throughout all of the cell-cycle phases (S, G1, or G2) (Supplementary Figs 3 and 4). Thus, Lnc_ASNR appear not to have any substantial effect on cell-cycle progress or arrest.

Next, we examined the effect of Lnc_ASNR on apoptosis. We first assessed the fraction of the cell population undergoing apoptosis by Annexin-V and FACS analysis. We observed a marked increase in the ratio of apoptotic cells after knockdown of Lnc_ASNR in RKO cell (Fig. 2C). We also observed an increase in Caspase 3 cleavage after knockdown of Lnc_ASNR (Fig. 2D).

In contrast, when overexpressing Lnc_ASNR in HCT116 cells (Fig. 1F) we observed strongly reduced binding of Annexin-V and increased cell proliferation (Fig. 2E). In addition, overexpression of Lnc_ASNR resulted in weaker Caspase 3 cleavage compared to control cells (Fig. 2F). Collectively, these results suggest that Lnc_ASNR promotes cell proliferation and suppresses cell apoptosis in cancer cell lines.

### Lnc_ASNR interacts with AUF1

Besides characterizing the function of Lnc_ASNR, we also sought to search in further detail the mechanism by which Lnc_ASNR down-regulate apoptosis. Many lncRNAs exert their functions through interacting with proteins, and we therefore, tried to identify possible protein partners of Lnc_ASNR, which might elucidate the molecular mechanisms by which Lnc_ASNR exerts its effects.

**Figure 3:**
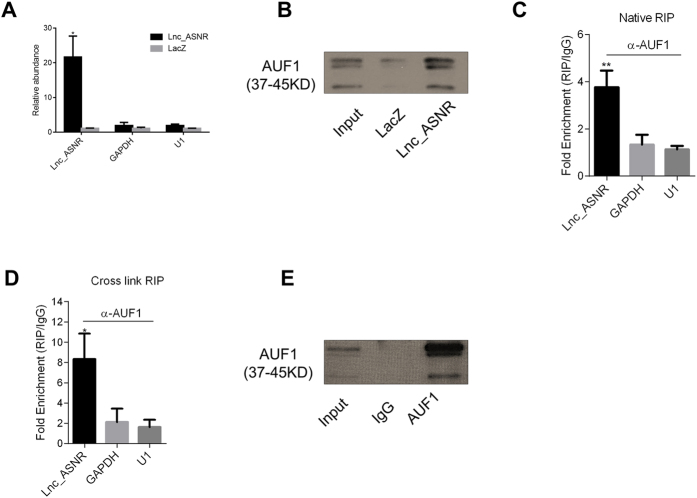
Lnc_ASNR interacts with AUF1. (**A**) Pull-down assay enriches Lnc_ASNR RNA 20-fold. All relative abundances were compared to 10% input. (**B**) Western blot of AUF1 pull down. The AUF1 antibody exhibits the four isoforms (p45, p42, p40, p37). (**C**) Native RNA immunoprecipitation (RIP) of AUF1 enriches Lnc_ASNR. U1 and GAPDH are negative controls. All relative abundances were compared to 10% input. (**D**) Formaldehyde cross-linked RNA immunoprecipitation (RIP) of AUF1 enriches Lnc_ASNR. U1 and GAPDH are negative controls. All relative abundances were compared to 10% input. (**E**) Western blot of AUF1 in the RIP assay.

RNA pull down with specific Lnc_ASNR antisense DNA probes resulted in more than 10 times enrichment of Lnc_ASNR compared to LacZ antisense DNA probes (Fig. 3A). The proteins associated with the pulled down Lnc_ANSR were separated on a Bis-Tris gel and the major band was excised and analyzed further by mass spectrometry assay in order to identify candidate associating proteins. The full gel image of pull-down sample was exhibited in supplementary data (Supplementary Fig. 5). The major protein identified was the heterogeneous nuclear ribonucleoprotein D (hnRNP D, also named as AUF1). We further analyzed the interaction between Lnc_ASNR and AUF1 by Western blot analysis that Lnc_ASNR could bind to all four isoforms of AUF1 (Fig. 3B).

To further validate the interaction between Lnc_ASNR and AUF1 in a cell-based systems, we obtained a commercial anti-AUF1 antibody with high specificity for AUF1 (Fig. 3E), which was used in a RNA immunoprecipitation (RIP) experiment on the nuclear fraction of RKO cells. The analysis showed an enrichment of Lnc_ASNR when using the AUF1 antibody as compared to using the nonspecific antibody (IgG control) (Fig. 3C). Cross-linking with formaldehyde RIP assay showed a higher enrichment of Lnc_ASNR (Fig. 3D).

Experimentally, the RNA pulldown method was employed to examine whether Lnc_ASNR can associate with AUF1 or not. Thus, we found that both the native and crosslink RIP could validate the interaction between Lnc_ASNR and AUF1 (Fig. 3C,D). Besides, it has been reported that AUF1 has three RNA binding motifs which dictate the RNA binding affinity^40^. Then we searched the motifs across the full length of Lnc_ASNR, and found several potential binding sites for each motif. Taken together, the long noncoding RNA, Lnc_ASNR, functions through interacting with AUF1 (Supplementary Fig. 6).

### Lnc_ASNR positively regulates Bcl-2

**Figure 4:**
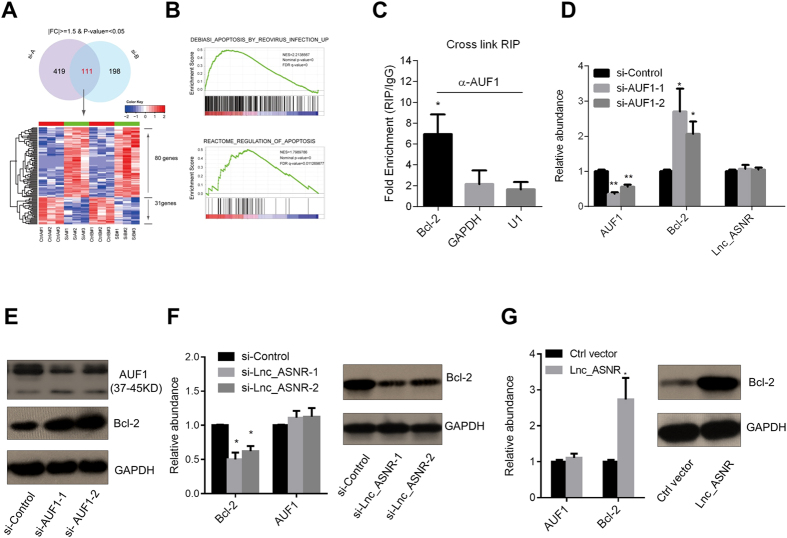
Lnc_ASNR positively regulates Bcl-2. (**A**) The Venn diagram indicates the 111 genes are expressionally changed in the two siRNAs mediated knockdown Lnc_ASNR (si-A represents si-Lnc_ASNR-1, and si-B represents si-Lnc_ASNR-2). The bottom panel show that the expression levels of overlapped 111genes (80 up-regulated and 31 down-regulated) were significantly altered in RKO cells when two independently siRNAs mediated knockdown of Lnc_ASNR (high expression: red, low expression: green). (**B**) The two representative gene sets (DEBIASI_APOPTOSIS_BY_REVIROUS_INFECTION_UP,REACTOME_REGUATION_OF_APOPTOSIS) of GSEA analysis are shown. (**C**) Formaldehyde cross-linked RNA immunoprecipitation (RIP) of AUF1 enriches Bcl-2. U1 and GAPDH are negative controls. All relative abundances were compared to 10% input. (**D**) Relative expression of AUF1, Bcl-2 and Lnc_ASNR after knock down of AUF1 by two different siRNAs (the two AUF1 siRNAs: siAUF1-1, siAUF1-2). (**E**) Western blot of AUF1 and Bcl-2 from D. (**F**) Relative expression of AUF1, Bcl-2 after knock down of Lnc_ASNR by two different siRNAs (F left). Western blot result of Bcl-2 in (F right). (**G**) Relative expression of Bcl-2 after overexpression of Lnc_ASNR (G left). Western blot result of Bcl-2 (G right).

We analyzed the transcriptome by microarray after knockdown of Lnc_ANSR and identified a set of apoptosis-related genes by GSEA (Fig. 4A,B) (NCBI GEO accession number GSE74568). Considering the evidence above that Lnc_ASNR suppress apoptosis, we next sought to explore if Lnc_ASNR and AUF1 function together to regulate specific genes related to this process. AUF1 has been shown to play a pro-apoptotic role by its ability to suppress the expression of the proto-oncogene Bcl-2^28^,^29^. As AUF1 and Lnc_ASNR both appear to regulate apoptosis, we sought to identify the operational relationship between these two genes. First, in order to demonstrate the interaction between AUF1 and Bcl-2 in our cell-based systems, we performed RNA immunoprecipitation (RIP) and observed an enrichment of Bcl-2 when using the AUF1 antibody (Fig. 4C). In addition, when we performed RNAi-mediated knockdown of AUF1 in RKO cell (using the non-targeting siRNA pool as a negative control), we observed a significant increase in the Bcl-2 mRNA level (Fig. 4D). The Western blot result was consistent with mRNA level (Fig. 4E).

**Figure 5:**
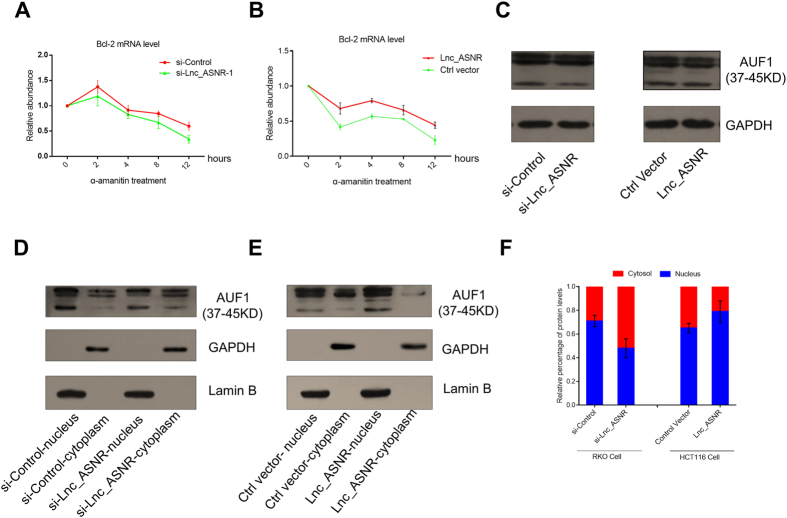
Lnc_ASNR suppresses Bcl-2 mRNA degradation through rising nucleus-retention of AUF1. (**A**) Relative abundance of Bcl-2 mRNA (qRT-PCR) cells treated with α-amanitin 48 h after Lnc_ASNR knockdown by siRNA transfection in RKO cells. Error bars represent standard deviation (±SD) in triplicate experiments. (**B**) Relative abundance of Bcl-2 mRNA (qRT-PCR) with α-amanitin treatment 24 h after transfection with the Lnc_ASNR expression vector pcDNA3.1 (+) in HCT116 cells. Error bars represent standard deviation (±SD) in triplicate experiments. (**C**) Western blots showing AUF1 protein expression in whole-cell lysates after knockdown (RKO cells) or overexpression (HCT116 cells) of Lnc_ASNR. (**D**) Western blots showing the distribution pattern of AUF1 protein in RKO cells after siRNA transfection. GAPDH and Lamin B1 were used as loading controls for proteins isolated from cytoplasmic and nuclear fractions, respectively. (**E**) Western blots showing the distribution pattern of AUF1 protein in RKO cells after pcDNA3.1 (+) Lnc_ASNR transfection. For GAPDH and Lamin B1 see D.

Analysis of Bcl-2 after knockdown of Lnc_ASNR showed that the expression level was reduced at both mRNA (qRT-PCR) and protein level (Western blot; Fig. 4F). In contrast, overexpression of Lnc_ASNR increased both mRNA and protein levels of Bcl-2 (Fig. 4G). However, AUF1 expression was apparently not affected by manipulation of the Lnc_ASNR levels (Fig. 5C, see also Fig. 4F,G).

Thus, a tentative working hypothesis might be that Lnc_ASNR increases the Bcl-2 expression level through interacting with AUF1.

### Lnc_ASNR suppresses Bcl-2 mRNA degradation through rising nucleus-retention of AUF1

The 3′UTR of Bcl-2 mRNA includes an extended AU-rich sequence that binds all four isoforms of AUF1, and which contribute to the Bcl-2 mRNA decay by conveying or recruiting additional trans-acting factors to the target mRNA for degradation^28^,^29^. We thus assumed that Lnc_ASNR may impact the Bcl-2 turnover rate. In order to investigate this hypothesis, we blocked mRNA synthesis by α-amanitin after 48 h knocked down of Lnc_ASNR with siRNAs, then analyzing the Bcl-2 mRNA levels at several time points. We observed that the Bcl-2 mRNA decay rate increased significantly after knockdown of Lnc_ASNR compared with the negative control (Fig. 5A). Conversely, in HCT116 cells overexpressing Lnc_ASNR, the Bcl-2 mRNA decay rate was reduced (Fig. 5B).

Nucleocytoplasmic shuttling is a common property of many RNA-binding proteins^37^. The subcellular distribution of AUF1 is dysregulated in diverse cancers modulated by cellular signaling pathways and other extrinsic stimuli^27^. Therefore, we assayed the fractions of the AUF1 protein located in nucleus and cytoplasm, in conjunction with Lnc_ASNR knockdown and overexpression. Knockdown of Lnc_ASNR increased the AUF1 levels in the cytoplasmic compartment (Fig. 5D), while overexpression of Lnc_ASNR led to more AUF1 being retained in the nucleus (Fig. 5E). Semiquantitation of the Western blot results by the Image J software is shown in Fig. 5F. In conclusion, we found that altering the Lnc_ASNR levels markedly changed the cytoplasmic/nuclear distribution of AUF1 without altering the overall cellular levels of AUF1 (Fig. 5C).

## Discussion

LncRNAs have emerged as important players in cellular development and human diseases^10^,^43^. LncRNA-based mechanisms control cell fates during development and they correlate with many human cancers caused by protein dysregulation^44^,^45^. Accumulating evidence suggests that lncRNAs contribute to tumor development and may serve as biomarkers and therapy targets. In this study, we have shown that the novel endogenous lncRNA Lnc_ASNR is highly expressed in all of the four different kinds of tumors tested compared with adjacent normal tissues. It is thus reasonable to surmise that the Lnc_ASNR RNA may play a role in the maintenance of cancer cell functions in these four tumor types. We have shown that Lnc_ASNR inhibits cell apoptosis. This function is consistent with an oncogenic role for Lnc_ASNR. For example, the overexpression of lncRNA SChLAP1 promoted cancer cell invasiveness and metastasis in malignant prostate tumors^46^. On the other hand, lncRNA PINT was down-regulated in primary colorectal tumors, thereby leading to increased proliferation of tumor cells^47^.

Recent bioinformatics surveys have indicated that as many as 5–8% of all genes contain ARE-like sequences in their 3′UTRs. Such ARE sequences are target positions for RNA binding proteins (RNPs)^22^,^36^, of which AUF1 is a well-known example. Among different mRNAs, AREs can vary widely in their sequence context. The Lnc_ASNR has similar sequences to which AUF1 potentially may bind (Supplementary Fig. 6). Our data showing that AUF1 cannot change the amount of Lnc_ASNR indicate that they may bind together and facilitate nucleus factors to participate in other biological process instead of accelerating the Lnc_ASNR degradation.

Our data suggests AUF1 bind to Bcl-2 mRNA in cytoplasmic compartment while Lnc_ASNR binds to AUF1 in the nucleus. Knockdown and overexpression of Lnc_ASNR changed the proportion of AUF1 in the cytoplasmic compartment. Accordingly, we made an assumption that Lnc_ASNR sequesters AUF1 in nucleus preventing its translocation into cytoplasm. Several studies have reported the AUF1 nucleocytoplasmic shuttling, but the mechanisms of AUFI nucleus-cytoplasm shuttle are contentious: Nuclear import of all AUF1 isoforms can bind transportin 1^48^. Normally, the two largest isoforms p42 and p45 are mainly nuclear forming hnRNP complex with pre-mRNA and scaffold attachment protein-β to anticipate in pre-mRNA splicing^49^,^50^,^51^, while p37 and p40 are disperse in both the nuclear and cytoplasmic compartments which can package into nmRNPs (mRNA-protein complexes) helping mRNA export or being sequestered into the cytoplasm by association with 14-3-3σ^50^,^52^. In addition, the translocation of AUF1 proteins altered in diverse cancers. For example, in MNT1 melanoma cells AUF1 is restricted to the nucleus, while normal melanocytes express some AUF1 proteins in the cytoplasm which are competent to bind ARE-containing mRNAs^38^. AUF1 does shuttle between nuclear and cytoplasmic compartments, but its subcellular distribution is highly isoform-dependent and can be modulated by cellular signaling pathways and other extrinsic stimuli (inflammation factors, nitric oxide exposure and hormonal signals), often coincident with altered expression of cancer-related products^27^. But, our Western blot experiment of AUF1 cell-fractionation cannot show dynamic processes of translocation of AUF1. The mechanism of lncRNAs being involved in the regulation of AUF1 nucleus/cytoplasmic compartment still need to be further explored. Some ideas could be used to explore the assumption: (1) we could test the variation of interaction between AUF1 and shuttle factors like transportin 1 and other export factors (AUF1-mRNA complex) after knockdown or overexpression of Lnc_ASNR. (2) Immunofluorescent visualization of AUF1 shuttling in/out of the nucleus could further investigate the translocation of AUF1 and visual interaction of AUF1 and Lnc_ASNR. (3) Lnc_ASNR changed the distribution of AUF1. Then, the location change of overexpressed fluorescence labeled AUF1 between knockdown and overexpression of Lnc_ASNR could be detected to investigate if Lnc_ASNR affects the translocation of AUF1. Further experiments are needed to clarify these possibilities.

Based on the above, we suggest a working model as follows: In normal cells, AUF1 binds to the Bcl-2 mRNA ARE region in the cytoplasm, which results in Bcl-2 mRNA decay. In normal cells, Lnc_ASNR may participate in other biological process. It is still a question to be explored (Supplementary Fig. 7).

**Figure 6:**
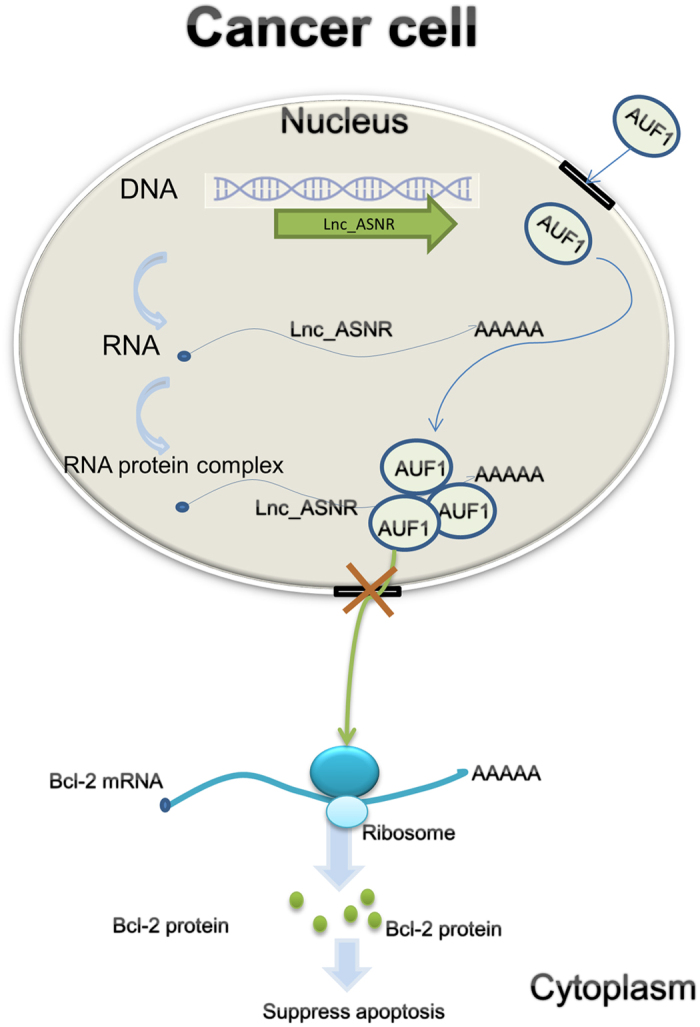
Proposed model for Lnc_ASNR action in cancer cells. In cancer cells, the AUF1 binds Lnc_ANSR in nucleus. As the nuclear retention of AUF1 is increased, cytoplasmic AUF1 levels decrease significantly, and as a result, more Bcl-2 protein is produced.

In cancer cells, as the nuclear retention of AUF1 is increased, cytoplasmic AUF1 levels decrease significantly, and as a result more Bcl-2 protein is produced (Fig. 6). In cancer cells, Lnc_ASNR/AUF1 complex may also bind to other factors participating in some cell specific process at multiple levels. It still needs further steps to elucidate the mechanism.

Through a combination of genomic, biochemical, and cell biological approaches, we concluded that formation of Lnc_ASNR-AUF1 complex in nucleus can decrease the cytoplasmic AUF1 to target Bcl-2 mRNA, resulting in increased production of Bcl-2 protein. It is becoming increasingly clear that mRNA stability is an important control point in the regulation of gene expression. Even though, AUF1 mediated degradation of Bcl-2 mRNA has been proved as one of the major mechanisms for Bcl-2 turnover^29^. The anti-apoptotic Bcl-2 protein is overexpressed in a variety of cancers. The Bcl-2 family of intracellular proteins is the central regulator of caspase activation, and its opposing factions of anti- and pro-apoptotic members arbitrate the life-or-death decision^53^. But, this data did not show that the effects observed must be mediated through AUF1 and Bcl-2. Such a hypothesis must be tested by simultaneous manipulation of ASNR, AUF1 and Bcl-2. Some experiments are still needed. (1) Simultaneous knockdown of Lnc_ASNR and AUF1 to detect Bcl-2 mRNA and protein level clarify whether Lnc_ASNR mediated AUF1 sublocation change occupies major factor for changed Bcl-2 level. (2) Rescue experiment of apoptosis after overexpression of Lnc_ASNR and knockdown of Bcl-2 could clarify whether Lnc_ASNR induced apoptosis is principally due to the variation in the amount of Bcl-2.

Meanwhile, our data does not exclude the possibility that the Lnc_ASNR induced apoptosis may be mediated by other unidentified cellular factors besides AUF1/Bcl-2 route. To clarify this concern, we will further explore the possibilities as below: First, microarrays of knockdown of Lnc_ASNR found a lot of differential expression genes which could give us more possibilities and clues that accounts for the oncogenetic role of Lnc_ASNR (Fig. 4A). In addition, the RNA pull down assay has defects in identifying the interacted proteins exactly. Methodological improvement over current methods to explore proteins interacting with Lnc_ASNR can be achieved when using CHIRP to capture proteins. In this method, crosslinked Lnc_ASNR and proteins reduced non-specific bindings and the enrichment could get to thousand times^54^.

In summary, lncRNA may point to new mechanisms of gene regulation, components in oncogenesis and potential targets for the development of molecular based cancer therapies.

## Materials and Methods

### Cell lines and culture conditions

The RKO and HCT116 cell lines were used in this study. RKO was cultured in MEM Medium (Life Technologies, 11095-080) with 10% Fetal Bovine Serum (FBS) (Life Technologies, 16000-044). HCT116 was cultured in McCoy’s 5A (Modified) Medium (Life Technologies, 16600-082) with 10% Fetal Bovine Serum (FBS).

### RNA extraction and qRT-PCR analyses

Total RNA was extracted from RKO or HCT116 cells with Trizol regent (Invitrogen, 15596-026) according to the manufacturer’s protocol, and DNA removed with recombinant DNase I (Ambion AM2235) enzyme. qRT-PCR was performed using the TransScript^®^ II Green One-Step qRT-PCR SuperMix (TransGen Biotech, AQ311-01) according to the manufacturer’s instructions. GAPDH was used as a positive control for mRNA. The primer sequences are listed in Supplementary Table 1.

### 5′ and 3′ RACE

5′-and 3′-RACE was performed using the RLM RACE kit (Ambion AM1700) as recommended by the manufacturer. The respective gene-specific primers are listed in Supplementary Table 1.

### Subcellular fractionation and Lamin B1, GAPDH measurement

The primer sequences are listed in Supplementary Table 1. Lamin B1 and GAPDH abundance were assessed with Western blot using their specific antibodies (Lamin B1, Proteintech, 66095-1-1g; GAPDH, Proteintech, 60004-1-1g). The procedure were described as previously^54^.

### Northern blot

Northern blots were performed using the Digoxigenin (DIG) Northern Starter Kit (Roche, 12039672910) according to the manufacturer’s protocol. The probes sequences are listed in Supplementary Table 1. The following hybridization and washing procedure were described as previously^55^.

### RNA interference

The transfection siRNA was performed with Lipofectamine 2000 (Invitrogen, 11668-019) according to the manufacturer’s protocol. Cells were digested by Trizol for RNA extraction after incubating 48 hours. siRNA sequences (5′ to 3′) for the knockdown experiments are listed in Supplementary Table 1.

### Cloning

All PCR primers used in this study are listed in Supplementary Table 1. The full-length Lnc_ASNR transcript was amplified from RKO cDNA and cloned into pcDNA3.1 (+) plasmid.

### Overexpression

Overexpression was performed using pcDNA3.1 (+) plasmid containing the transcript sequence while empty plasmid served as negative control. The transfection of plasmid was performed with Lipo3000 Transfection Reagent (Invitrogen, L3000-008) according to the manufacturer’s protocol. The efficiency of the overexpression was determined by qRT-PCR or Western blot.

### Cell proliferation assay

Cell proliferation was tested using the CellTiter 96AQueous One Solution Cell Proliferation Assay (MTS) (Promega, G3582). The transfected cells were plated in 96-well plates (2000 cells/well). Cell proliferation was determined every 24 hours following the manufacturer’s protocol.

### Cell apoptosis assay and Cleaved Caspase-3 measurement

For cell apoptosis quantification, cell viability was determined by staining with Annexin V, Alexa Fluor 488 conjugate (Life Technologies, A13201) and propidium iodide (Life Technologies, P21493) and detected by flow cytometry (BD FACSVERSE) according to the Annexin V flow cytometry protocol. Western blot of the Cleaved Caspase-3 antibody (ABGENT, AP3725a) was measured for the Caspace-3 abundance. The cells were collected for flow cytometry assays or total protein lysate 48 h after transfection.

### RNA-pulldown

Antisense DNA probes were designed against Lnc_ASNR and LacZ RNA as controls (http://www.singlemoleculefish.com). Nine probes were generated against full length of Lnc_ASNR and eight for LacZ. All probes were biotinylated at the 3′ end Eight 15 cm plates of RKO cells were lysed in cytoplasm isolation buffer (5xcytoplasm-isolationbuffer: 1.28 M sucrose, 40 Mm Tris-HCl (PH7.5), 20 mM MgCL2, 4%Triton-100)on ice for 20 min, and centrifuged for 2500 g 20 min. The supernatant was discarded and nuclear sediment was dissolved in RIP buffer (150 mM KCL, 25 mM Tris PH7.4, 0.5 Mm DTT, 5 Mm EDTA, 0.5% NP40) for 20 min and vortexed fiercely. The solution was centrifuged at 13000 g for 10 min and the soluble lysate retained. The lysate was divided into two equal aliquots, one for probes targeting the lncRNA, and the other for probes targeting LacZ as controls. For each aliquot, we used 600 pmol probes and 240 μl streptavidin magnetic C1 beads. Probes and beads were incubated with cell lysate and the beads were washed for 5 times with RIP buffer. We separated the beads into two parts, 4/5 for RNA elution and 1/5 for protein elution. RNA was extracted with the Trizol reagent. qRT-PCR was performed to determine the enrichment of target RNAs. The proteins were separated on a 4–12% Bis-Tris Gel (Life Technologies, NP0335BOX) and stained with the Silver Staining Kit (Life Technologies, LC6070) and analyzed by mass spectrometry (MS). The probe sequences are listed in Supplementary Table 1.

### Native RNA immunoprecipitation (RIP) and cross- linking RIP

The RIP experiments were performed using the Pierce protein A/G Magnetic Beads (Thermo, 88803) according to the manufacturer’s instructions. 10% the RIP product and 10% the cell lysate input were used for Western blot as described above, while rest of the RIP product was used for RIP qRT-PCR. The AUF1 antibody (Merck Millipore, 03-111) was used for the RIP assays. All RIP assays were performed in biological duplicate. The formaldehyde crosslink RIP was performed as described^56^.

### α-amanitin treatment

The transfected cells were plated in 12-well plates for 24–48 hours. Then, 8 μl (1.0 mg/mL) α-amanitin was added to every well in the 12-well plates following the manufacturer’s protocol (α-amanitin, Sigma, A2263-1MG). Each well has 1 ml solution. qRT-PCR was performed to detect the specific mRNA level after 0, 2, 4, 8, 12 hours drug administration.

### Statistical Analysis

Statistical analyses were performed on GraphPad Prism Software (California, US). The two-tailed student’s *t* test was used to compare variables between the two groups. Bars indicate SEM. *P < 0.05, **P < 0.01, ***P < 0.001 (throughout all figures). All experiments were repeated three times if no specially indicated.

## Additional Information

**How to cite this article**: Chen, J. *et al*. The long noncoding RNA ASNR regulates degradation of Bcl-2 mRNA through its interaction with AUF1. *Sci. Rep.* **6**, 32189; doi: 10.1038/srep32189 (2016).

## Supplementary Material

Supplementary Information
