# Supplementary material for: The long noncoding RNA ASNR regulates degradation of Bcl-2 mRNA through its interaction with AUF1
Source: Sci Rep. 2016 Aug 31;6:32189. doi: 10.1038/srep32189 (PMC5006016; doi:10.1038/srep32189)
Supplement: Supplementary Information [file srep32189-s1.pdf]

Title: The long noncoding RNA ASNR regulates degradation of Bcl-2 mRNA through its interaction with AUF1

Authors: Jiahui Chen[1,2,3], Lihui Liu[1,2,3], Guifeng Wei[1,2,3], Wei Wu[1,2,3], Huaxia Luo[1,2,3], Jiao Yuan[1,2,3],Jianjun Luo[1,2], and Runsheng Chen[1,2,4]*

Affiliations:

[1]Key Laboratory of RNA Biology, Institute of Biophysics, Chinese Academy of Sciences, Beijing 100101, China

[2]Beijing Key Laboratory of Noncoding RNA, Institute of Biophysics, Chinese Academy of Sciences, Beijing 100101, China

[3]University of Chinese Academy of Sciences, Beijing 100049, China

[4]Research Network of Computational Biology, RNCB, Beijing, 100101, China

* Correspondence to:

Runsheng Chen (+86-10-64888546, rschen@ibp.ac.cn)

This PDF file includes:

Supplementary Figures 1-7 and Supplementary Table.

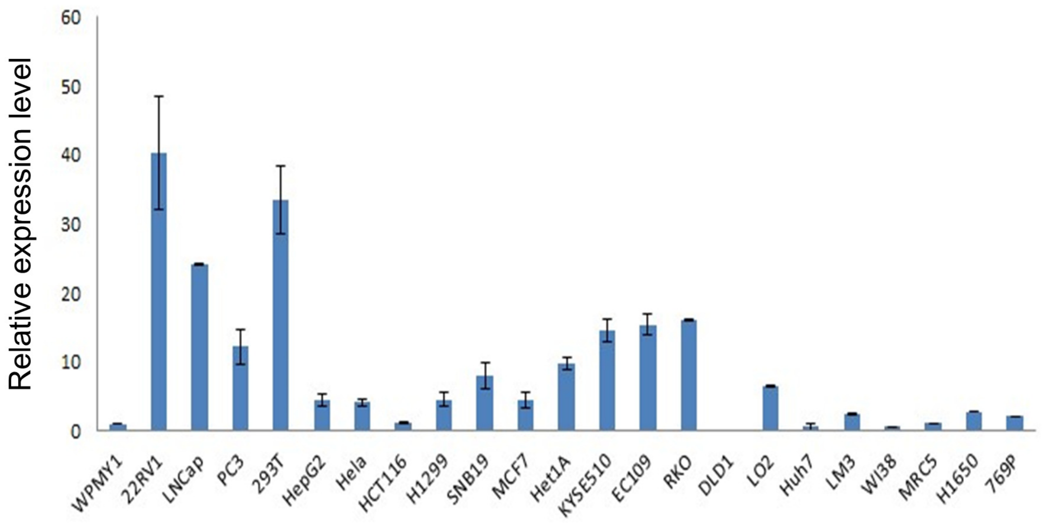

Supplementary Fig.1 Relative expression of Lnc_ASNR in different cells

Quantification of Lnc_ASNR expression in 23 kinds of cell lysates by qRT-PCR and GAPDH serve as controls for gene expression. Error bars represent SEM, n=3.

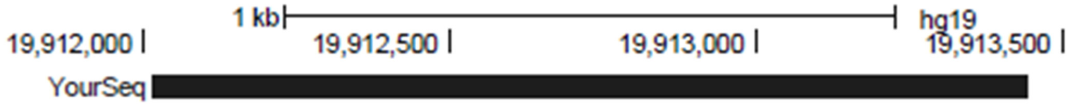

Chr14 >19913257-19912166

Supplementary Fig.2 3’ RACE and 5’ RACE of Lnc_ASNR.

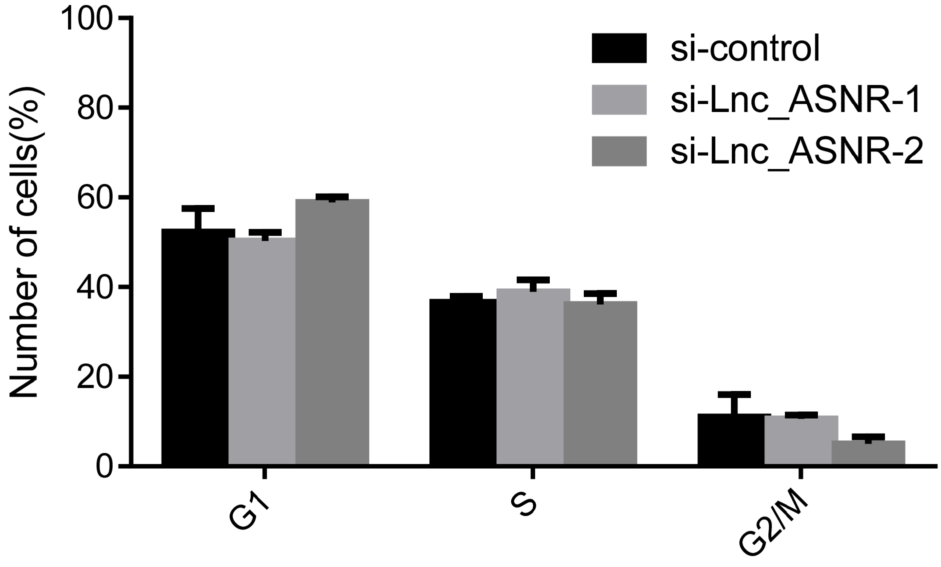

Supplementary Fig.3 Lnc_ASNR knockdown doesn't affect cell cycle regulation.

Cell cycle results of Lnc_ASNR knockdown were measured by flow cytometry. The X-axis represents cell cycle phases. The Y-axis gives the results from quantification (% cells) average of three independent replicate experiments. Bars indicate SEM.

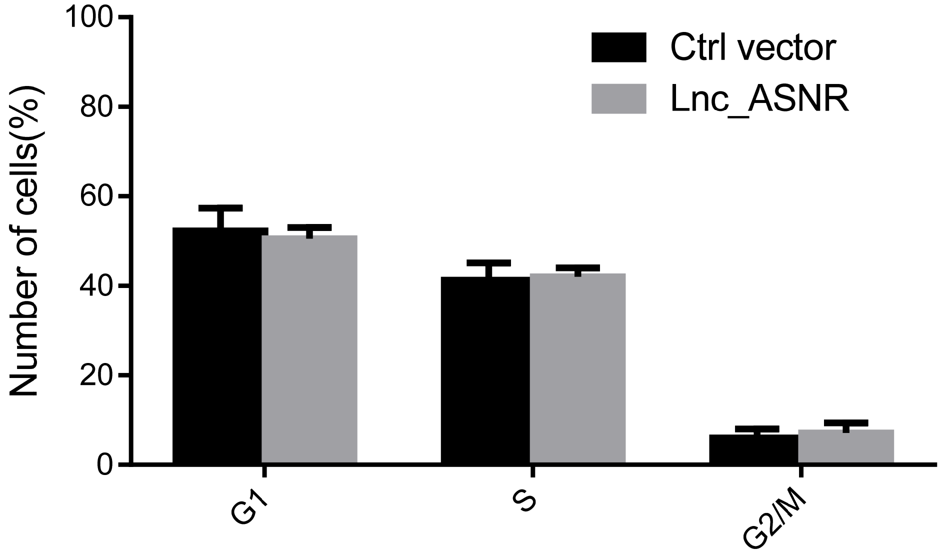

Supplementary Fig.4 Overexpression of Lnc_ASNR doesn't affect cell cycle regulation.

Cell cycle results of Lnc_ASNR overexpression were measured by flow cytometry. The X-axis represents cell cycle phases. The Y-axis gives the results from quantification (% cells) average of three independent replicate experiments. Bars indicate SEM.

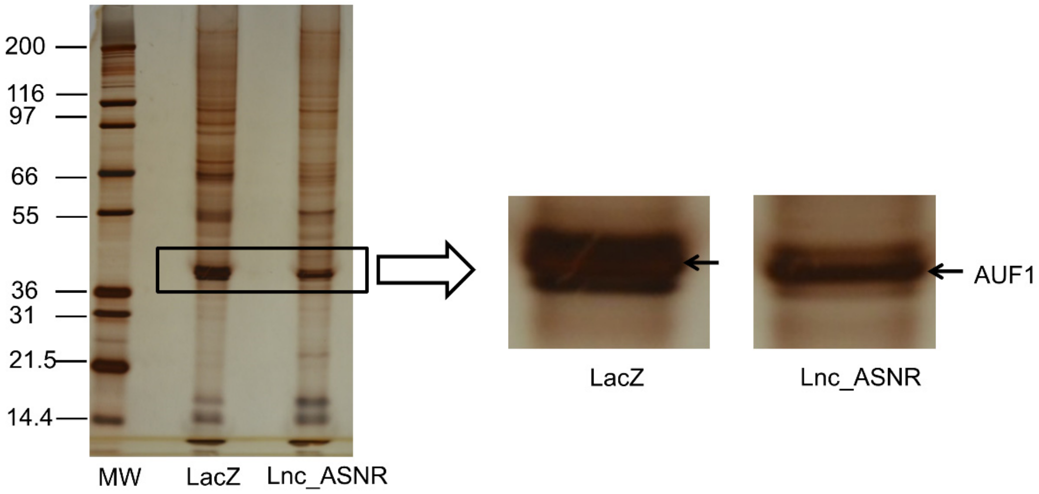

Supplementary Fig.5 Silver stains gel of RNA-pulldown.

SDS-PAGE gel of proteins pulled down by the Lnc_ASNR and LacZ probes. Band indicated by arrows were submitted for mass spectrometry.

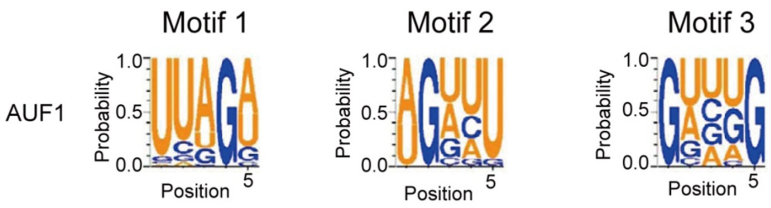

Motif 1 :

No	start	end
1	19912323	19912327
2	19912585	19912589

Motif 2 :

No	start	end
1	19912290	19912294
2	19912398	19912402
3	19912578	19912582
4	19912695	19912699
5	19912855	19912859
6	19913578	19913582
7	19913585	19913589

Motif 3 :

No	start	end
1	19912399	19912403
2	19912579	19912583
3	19913579	19913583

Example : Site 2 of motif 1

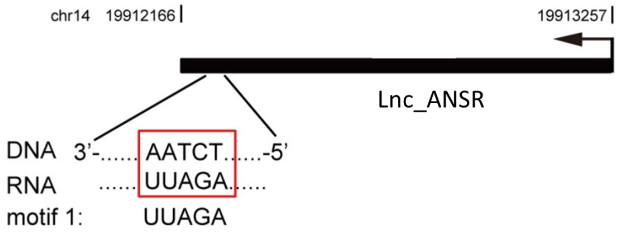

Supplementary Fig.6 Computational search the binding motifs of AUF1 across the full-length of Lnc_ASNR revealed the potential binding sites.

The binding motifs of AUF1 information from published reference. The binding sites across the full length of Lnc_ASNR were shown on the chromatin location. Example of binding position (UUAGA) of AUF1 showed that AUF1 binds to sequence UUAGA of Lnc_ASNR.

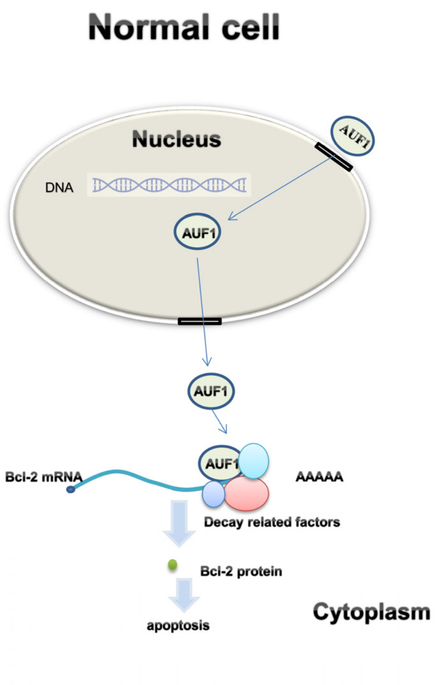

Supplementary Fig.7 The reported model of AUF1 mediated Blc-2 degradation in normal cell.

In normal cells, AUF1 binds to the Bcl-2 mRNA ARE region in the cytoplasm, which resulting in Bcl-2 mRNA decay.

Supplementary Table

RACE primers

Lnc_ASNR-5'RACE-R1	5' GAAGAACAACAAATAAGACTTAAGAGGAAAAGGAAT 3'
Lnc_ASNR-5'RACE-R2	5' TGCAGCCTACTAAATCAGAATGAAAATAGA 3'
Lnc_ASNR-3'RACE-F1	5' AGTTCCTGCTTTCTTGTCAAAAATTGAAG 3'
Lnc_ASNR-3'RACE-F2	5' CTTCTAGGAACCCATCATCCAAGAC 3'

Northern Blotting Probe Primers

Lnc_ASNR LNA probe: /5DigN/TAATGGTGTAAGCAATGTGGAT the probe designed from exiqon webset

hGAPDH_F	GGAAGGTGAAGGTCGGAGT	capital nucleotides indicated T7 promoter sequences
hGAPDH_T7_R	agtcatTAATACGACTCACTATAGGAAGGGGTGCTAAGCAGTTGGT	

qPCR primers

Lnc_ASNR_q_F	GAGAATTCCTCTGGTGGCTAAA
Lnc_ASNR_q_R	GGAACTGTAATCTCACTGGACAA
AUF1-q-F	ATGTCGGAGGAGCAGTTCGGCGGGGAC
AUF1-q-R	TTAGTATGGTTTGTAGCTATTTTGATG
Bcl-2-q-F	GGAGGATTGTGGCCTTCTTT
Bcl-2-q-R	GTTCAGGTACTCAGTCATCCAC
linc01296-Qpcr-F	TAATGATCACCTGCGCCTTC
linc01296-Qpcr-R	AGCAACCATCGCCCTTATC
linc01296.1-Qpcr-F	CATAGGTCACCAGCTTCACTTC
linc01296.1-Qpcr-R	CACTCCATACCTTGCTCAACTAC
BMSF18/17-Qpcr-F	GAAACCAGTGGGTGAGAAAGA
BMSF18/17-Qpcr-R	CAGGCTGCATCTCCACTAATAC
DUXAP10-Qpcr-F	GCAAGGTTTCTGAGGGACTT
DUXAP10-Qpcr-R	TCACCATGTTCTGGCTCTTG
GAPDH_q_F	TGCCATCAATGACCCCTTC
GAPDH_q_R	CATCGCCCCACTTGATTTTG
U1_q_F	GGGAGATACCATGATCACGAAGGT
U1_q_R	CCACAAATTATGCAGTCGAGTTTCCC
β-actin_q_F	ATTTAAAAACTGGAACGGTGAAGGT
β-actin_q_R	CTGTAACAACGCATCTCATATTTGG

Construct primers

Lnc_ASNR-F	5' GG GGTACC CAGGAAACTCCAAACTGTCCAAGGA 3'
Lnc_ASNR-R	5' CG GGATCC GTAACAAACTTTATTTTTGATGAGTGAAGACT 3'

siRNAs

	sense(5'-3')	antisense（5'-3'）
si-Lnc_ASNR-1	rGrGrArGrArArUrGrCrArGrArArUrGrGrUrCrArGrArCrUAC	rGrGrArGrArArUrGrCrArGrArArUrGrGrUrCrArGrArCrUAC
si-Lnc_ASNR-2	rGrCrArGrGrArArCrUrUrGrArUrUrArCrUrUrUrGrArGrUGC	rGrCrArCrUrCrArArArGrUrArArUrCrArArGrUrUrCrCrUrGrCrUrU
si-Lnc_ASNR-3	rArCrArCrCrArUrUrArGrGrCrArUrArGrArUrUrCrArGrUGT	rArCrArCrUrGrArArUrCrUrArUrGrCrCrUrArArUrGrGrUrGrUrArA
si-AUF1-1	rCrCrArCrArArUrGrUrUrGrGrUrCrUrUrArGrUrArArArUGT	rArCrArUrUrUrArCrUrArArGrArCrCrArArCrArUrUrGrUrGrGrUrA
si-AUF1-2	rArCrArArUrArUrCrArGrCrArArCrArGrCrArArCrArGrUGG	rCrCrArCrUrGrUrUrGrCrUrGrUrUrGrCrUrGrArUrArUrUrGrUrUrC

r represents ribonucleic acid.

Pulldown 3' biotinylated probes

Target	Probe Name	Sequence of Oligo (5'- to -3')
**Lnc_ASNR**	ASNR_p1	caacagaactatctccttgg
	ASNR_p2	cacctgagattttgctctac
	ASNR_p3	ccaagactgcggcactaaaa
	ASNR_p4	ctgtgacagtggcagaagaa
	ASNR_p5	ctcaagatgaaagactccct
	ASNR_p6	gcagaatggtcagactacca
	ASNR_p7	ctatgcctaatggtgtaagc
	ASNR_p8	atctggatcaagcagtttgg
	ASNR_p9	gtgtgcagcctactaaatcag
**Non-Targetting Control (LacZ)**	lacZ_p1	GCCAGTGAATCCGTAATCAT
	lacZ_p2	AATGTGAGCGAGTAACAACC
	lacZ_p3	GATCTTCCAGATAACTGCCG
	lacZ_p4	GCTCATCGATAATTTCACCG
	lacZ_p5	TTAACGCCTCGAATCAGCAA
	lacZ_p6	ATTTGATCCAGCGATACAGC
	lacZ_p7	GGGTTGCCGTTTTCATCATA
	lacZ_p8	CACTTACGCCAATGTCGTTA
